# Project TENDR: Targeting Environmental Neuro-Developmental Risks The TENDR Consensus Statement

**DOI:** 10.1289/EHP358

**Published:** 2016-07-01

**Authors:** Deborah Bennett, David C. Bellinger, Linda S. Birnbaum, DABT, A.T.S, Asa Bradman, Aimin Chen, Deborah A. Cory-Slechta, Stephanie M. Engel, M. Daniele Fallin, Alycia Halladay, Russ Hauser, Irva Hertz-Picciotto, Carol F. Kwiatkowski, Bruce P. Lanphear, Emily Marquez, Melanie Marty, Jennifer McPartland, Craig J. Newschaffer, Devon Payne-Sturges, Heather B. Patisaul, Frederica P. Perera, Beate Ritz, Jennifer Sass, Susan L. Schantz, Thomas F. Webster, Robin M. Whyatt, Tracey J. Woodruff, R. Thomas Zoeller, Laura Anderko, Carla Campbell, Jeanne A. Conry, Nathaniel DeNicola, Robert M. Gould, Deborah Hirtz, Katie Huffling, Philip J. Landrigan, Arthur Lavin, Mark Miller, Mark A. Mitchell, Leslie Rubin, Ted Schettler, Ho Luong Tran, Annie Acosta, Charlotte Brody, Elise Miller, Pamela Miller, Maureen Swanson, Nsedu Obot Witherspoon, American College of Obstetricians and Gynecologists (ACOG), Child Neurology Society, Endocrine Society, International Neurotoxicology Association, International Society for Children's Health and the Environment, International Society for Environmental Epidemiology, National Council of Asian Pacific Islander Physicians, National Hispanic Medical Association, National Medical Association

**Affiliations:** Associate Professor, Department of Public Health Sciences, School of Medicine,; University of California, Davis; Boston Children's Hospital; Harvard Medical School; Harvard T.H. Chan School of Public Health; Director, National Institute of Environmental Health Sciences,; Director, National Toxicology Program; Associate Director, Center for Environmental Research and Children's Health (CERCH); Associate Adjunct Professor of Environmental Health Sciences, School of Public Health, UC Berkeley; Associate Professor, Division of Epidemiology; Department of Environmental Health; University of Cincinnati College of Medicine; Professor of Environmental Medicine, Pediatrics and Public Health Sciences; Acting Chair, Department of Environmental Medicine;; Director, University of Rochester Medical School Environmental Health Sciences Center; Associate Professor, Department of Epidemiology,; Gillings School of Global Public Health University of North Carolina, Chapel Hill; Sylvia and Harold Halpert Professor and Chair,; Dept. of Mental Health; Director, Wendy Klag Center for Autism and Developmental Disabilities, Johns Hopkins Bloomberg School of Public Health; Chief Science Officer, Autism Science Foundation, Adjunct, Dept. of Pharmacology and Toxicology,; Rutgers University; Frederick Lee Hisaw Professor of Reproductive Physiology; Professor of Environmental and Occupational Epidemiology, Harvard T.H. Chan School of Public Health; Professor of Obstetrics, Gynecology and Reproductive Biology, Harvard Medical School; Director, UC Davis Environmental Health Sciences Center; Professor, Department of Public Health Sciences & Medical Investigations of Neurodevelopmental Disorders (MIND) Institute,; University of California, Davis; Executive Director, The Endocrine Disruption Exchange (TEDX); Assistant Professor Adjunct, Dept. of Integrative Physiology, University of Colorado, Boulder; Clinician Scientist, Child & Family Research Institute, BC Children's Hospital Professor, Faculty of Health Sciences, Simon Fraser University, Vancouver, BC; Staff Scientist; Pesticide Action Network North America; Adjunct Associate Professor; University of California, Davis; Senior Scientist; Environmental Defense Fund; Director, A.J. Drexel Autism Institute; Professor, Epidemiology and Biostatistics,; Drexel University; Assistant Professor, Maryland Institute for Applied Environmental Health, School of Public Health,; University of Maryland; Professor, Biological Sciences, Center for Human Health and the Environment, WM Keck Center for Behavioral Biology, NC State University; Professor of Public Health; Director, Columbia Center for Children's Environmental Health; Professor, Dept. of Environmental Health Sciences, Mailman School of Public Health, Columbia University; Professor of Epidemiology; Center for Occupational and Environmental Health; Fielding School of Public Health, University of California Los Angeles; Senior Scientist, Natural Resources Defense Council; Professorial Lecturer, George Washington University; Professor of Toxicology and Neuroscience, Illinois Children's Environmental Health Research Center; Director, Beckman Institute for Advanced Science and Technology, University of Illinois, Urbana-Champaign; Professor, Department of Environmental Health, Boston University School of Public Health; Professor Emeritus, Department of Environmental Health Sciences, Mailman School of Public Health, Columbia University; Professor and Director, Program on Reproductive Health and the Environment, Dept. of Obstetrics, Gynecology and Reproductive Sciences, University of California, San Francisco; Professor of Biology; Director, Laboratory of Molecular & Cellular Biology,; University of Massachusetts Amherst; Robert and Kathleen Scanlon Endowed Chair in Values Based Health Care & Professor, School of Nursing and Health Studies, Georgetown University,; Visiting Clinical Associate Professor of Public Health University of Texas at El Paso; Past President, American College of Obstetricians and Gynecologists; Assistant Physician in Chief, The Permanente Medical Group; American College of Obstetricians & Gynecologists Liaison to American Academy of Pediatrics Executive Council on Environmental Health; Clinical Associate in Obstetrics & Gynecology, U. of Pennsylvania; Associate Adjunct Professor, Program on Reproductive Health and the Environment,; Dept. of Obstetrics, Gynecology and Reproductive Sciences, UCSF School of Medicine; Immediate Past President, Physicians for Social Responsibility; Professor, Neurological Sciences and Pediatrics; University of Vermont School of Medicine; Director of Programs; Alliance of Nurses for Healthy Environments; Dean for Global Health, Arnhold Institute for Global Health; Professor of Preventive Medicine and Pediatrics, Icahn School of Medicine at Mount Sinai; Advanced Pediatrics; Associate Clinical Professor of Pediatrics; Case Western Reserve University School of Medicine; Director, University of California San Francisco Pediatric Environmental Health Specialty Unit; President, Mitchell Environmental Health Associates; Chair, Council on Medical Legislation and; Co-Chair, Commission on Environmental Health; National Medical Association; President, Innovative Solutions for Disadvantage and Disability; Associate Professor, Dept. of Pediatrics, Morehouse School of Medicine; Co-director, Southeast Pediatric Environmental Health Specialty Unit, Emory University; Medical Director, Developmental Pediatric Specialists; Science Director; Science and Environmental Health Network; President and CEO; National Council of Asian Pacific Islander Physicians; Director of Fiscal and Family Support Policy; The Arc; National Coordinator, Healthy Babies Bright Futures Vice President of Health Initiatives, BlueGreen Alliance; Director, Collaborative on Health and the Environment (CHE); Executive Director; Alaska Community Action on Toxics; Healthy Children Project Director; Learning Disabilities Association of America; Executive Director, Children's Environmental Health Network

## Abstract

**Summary: **Children in America today are at an unacceptably high risk of developing neurodevelopmental disorders that affect the brain and nervous system including autism, attention deficit hyperactivity disorder, intellectual disabilities, and other learning and behavioral disabilities. These are complex disorders with multiple causes—genetic, social, and environmental. The contribution of toxic chemicals to these disorders can be prevented. **Approach:** Leading scientific and medical experts, along with children’s health advocates, came together in 2015 under the auspices of Project TENDR: Targeting Environmental Neuro-Developmental Risks to issue a call to action to reduce widespread exposures to chemicals that interfere with fetal and children’s brain development. Based on the available scientific evidence, the TENDR authors have identified prime examples of toxic chemicals and pollutants that increase children’s risks for neurodevelopmental disorders. These include chemicals that are used extensively in consumer products and that have become widespread in the environment. Some are chemicals to which children and pregnant women are regularly exposed, and they are detected in the bodies of virtually all Americans in national surveys conducted by the U.S. Centers for Disease Control and Prevention. The vast majority of chemicals in industrial and consumer products undergo almost no testing for developmental neurotoxicity or other health effects. **Conclusion: **Based on these findings, we assert that the current system in the United States for evaluating scientific evidence and making health-based decisions about environmental chemicals is fundamentally broken. To help reduce the unacceptably high prevalence of neurodevelopmental disorders in our children, we must eliminate or significantly reduce exposures to chemicals that contribute to these conditions. We must adopt a new framework for assessing chemicals that have the potential to disrupt brain development and prevent the use of those that may pose a risk. This consensus statement lays the foundation for developing recommendations to monitor, assess, and reduce exposures to neurotoxic chemicals. These measures are urgently needed if we are to protect healthy brain development so that current and future generations can reach their fullest potential.

## A Call to Action

The TENDR Consensus Statement is a call to action to reduce exposures to toxic chemicals that can contribute to the prevalence of neurodevelopmental disabilities in America’s children. The TENDR authors agree that widespread exposures to toxic chemicals in our air, water, food, soil, and consumer products can increase the risks for cognitive, behavioral, or social impairment, as well as specific neurodevelopmental disorders such as autism and attention deficit hyperactivity disorder (ADHD) (Di Renzo et al. 2015; Gore et al. 2015; Lanphear 2015; Council on Environmental Health 2011). This preventable threat results from a failure of our industrial and consumer markets and regulatory systems to protect the developing brain from toxic chemicals. To lower children’s risks for developing neurodevelopmental disorders, policies and actions are urgently needed to eliminate or significantly reduce exposures to these chemicals. Further, if we are to protect children, we must overhaul how government agencies and business assess risks to human health from chemical exposures, how chemicals in commerce are regulated, and how scientific evidence informs decision making by government and the private sector.

### Trends in Neurodevelopmental Disorders

We are witnessing an alarming increase in learning and behavioral problems in children. Parents report that 1 in 6 children in the United States, 17% more than a decade ago, have a developmental disability, including learning disabilities, ADHD, autism, and other developmental delays (Boyle et al. 2011). As of 2012, 1 in 10 (> 5.9 million) children in the United States are estimated to have ADHD (Bloom et al. 2013). As of 2014, 1 in 68 children in the United States has an autism spectrum disorder (based on 2010 reporting data) (CDC 2014).

The economic costs associated with neurodevelopmental disorders are staggering. On average, it costs twice as much in the United States to educate a child who has a learning or developmental disability as it costs for a child who does not (Chambers et al. 2004). A recent study in the European Union found that costs associated with lost IQ points and intellectual disability arising from two categories of chemicals—polybrominated diphenyl ether flame retardants (PBDEs) and organophosphate (OP) pesticides—are estimated at 155.44 billion euros ($169.43 billion dollars) annually (Bellanger et al. 2015). A 2009 analysis in the United States found that for every $1 spent to reduce exposures to lead, a potent neurotoxicant, society would benefit by $17–$221 (Gould 2009).

### Vulnerability of the Developing Brain to Chemicals

Many toxic chemicals can interfere with healthy brain development, some at extremely low levels of exposure (Adamkiewicz et al. 2011; Bellinger 2008; Committee on Improving Analysis Approaches Used by the U.S. EPA 2009; Zoeller et al. 2012). Research in the neurosciences has identified “critical windows of vulnerability” during embryonic and fetal development, infancy, early childhood and adolescence (Lanphear 2015; Lyall et al. 2014; Rice and Barone 2000). During these windows of development, toxic chemical exposures may cause lasting harm to the brain that interferes with a child’s ability to reach his or her full potential.

The developing fetus is continuously exposed to a mixture of environmental chemicals (Mitro et al. 2015). A 2011 analysis of the U.S. Centers for Disease Control and Prevention’s (CDC) biomonitoring data found that 90% of pregnant women in the United States have detectable levels of 62 chemicals in their bodies, out of 163 chemicals for which the women were screened (Woodruff et al. 2011). Among the chemicals found in the vast majority of pregnant women are PBDEs, polycyclic aromatic hydrocarbons (PAHS), phthalates, perfluorinated compounds, polychlorinated biphenyls (PCBs), perchlorate, lead and mercury (Woodruff et al. 2011). Many of these chemicals can cross the placenta during pregnancy and are routinely detected in cord blood or other fetal tissues (ATSDR 2011; Brent 2010; Chen et al. 2013; Lien et al. 2011).

### Prime Examples of Neurodevelopmentally Toxic Chemicals

The following list provides prime examples of toxic chemicals that can contribute to learning, behavioral, or intellectual impairment, as well as specific neurodevelopmental disorders such as ADHD or autism spectrum disorder:

Organophosphate (OP) pesticides (Eskenazi et al. 2007; Fortenberry et al. 2014; Furlong et al. 2014; Marks et al. 2010; Rauh et al. 2006; Shelton et al. 2014).
PBDE flame retardants (Chen et al. 2014; Cowell et al. 2015; Eskenazi et al. 2013; Herbstman et al. 2010).
Combustion-related air pollutants, which generally include PAHs, nitrogen dioxide and particulate matter, and other air pollutants for which nitrogen dioxide and particulate matter are markers (Becerra et al. 2013; Clifford et al. 2016; Jedrychowski et al. 2015; Kalkbrenner et al. 2014; Suades-González et al. 2015; Volk et al. 2013).
Lead (Eubig et al. 2010; Lanphear et al. 2005; Needleman et al. 1979).
Mercury (Grandjean et al. 1997; Karagas et al. 2012; Sagiv et al. 2012).
PCBs (Eubig et al. 2010; Jacobson and Jacobson 1996; Schantz et al. 2003).

The United States has restricted some of the production, use and environmental releases of these particular chemicals, but those measures have tended to be too little and too late. We face a crisis from both legacy and ongoing exposures to toxic chemicals. For lead, OP pesticides, PBDEs and air pollution, communities of color and socioeconomically stressed communities face disproportionately high exposures and health impacts (Adamkiewicz et al. 2011; Engel et al. 2015; Zota et al. 2010).

Policies to ban lead from gasoline, paints and other products have been successful in lowering blood lead levels in the American population (Jones et al. 2009), yet lead exposure continues to be a preventable cause of intellectual impairment, ADHD and maladaptive behaviors for millions of children (CDC 2015). Scientists agree that there is no safe level of lead exposure for fetal or early childhood development (Lanphear et al. 2005; Schnur and John 2014), and studies have documented the potential for cumulative and synergistic health effects from combined exposure to lead and social stressors (Bellinger et al. 1988; Cory-Slechta et al. 2004). Thus, taking further preventive actions is imperative.

Epidemiological, toxicological, and mechanistic studies have together provided evidence that clearly demonstrates or strongly suggests neurodevelopmental toxicity for lead, mercury, OP pesticides, air pollution, PBDEs, and PCBs. The level and type of available evidence linking exposures to toxic chemicals with neurodevelopmental disorders, including the examples in this statement, vary both within and among chemical classes. In light of this extensive evidence and continued widespread exposure, the risks for learning and developmental disorders can likely be lowered through targeted exposure reduction, starting with these example chemicals.

### Majority of Chemicals Untested for Neurodevelopmental Effects

The examples of developmental neurotoxic chemicals that we list here likely represent the tip of the iceberg. Of the tens of thousands of chemicals on the U.S. Environmental Protection Agency (EPA) chemical inventory, nearly 7,700 are manufactured or imported into the United States at ≥ 25,000 pounds per year (U.S. EPA 2012). The U.S. EPA has identified nearly 3,000 chemicals that are produced or imported at > 1 million pounds per year (U.S. EPA 2006).

Only a minority of chemicals has been evaluated for neurotoxic effects in adults. Even fewer have been evaluated for potential effects on brain development in children (Grandjean and Landrigan 2006, 2014). Further, toxicological studies and regulatory evaluation seldom address combined effects of chemical mixtures, despite evidence that all people are exposed to dozens of chemicals at any given time.

### Need for a New Approach to Evaluating Evidence

Our failures to protect children from harm underscore the urgent need for a better approach to developing and assessing scientific evidence and using it to make decisions. We as a society should be able to take protective action when scientific evidence indicates a chemical is of concern, and not wait for unequivocal proof that a chemical is causing harm to our children.

Evidence of neurodevelopmental toxicity of any type—epidemiological or toxicological or mechanistic—by itself should constitute a signal sufficient to trigger prioritization and some level of action. Such an approach would enable policy makers and regulators to proactively test and identify chemicals that are emerging concerns for brain development and prevent widespread human exposures.

Some chemicals, like those that disrupt the endocrine system, present a concern because they interfere with the activity of endogenous hormones that are essential for healthy brain development. Endocrine-disrupting chemicals (EDCs) include many pesticides, flame retardants, fuels, and plasticizers. One class of EDCs that is ubiquitous in consumer products are the phthalates. These are an emerging concern for interference with brain development and therefore demand attention (Boas et al. 2012; Ejaredar et al. 2015; Mathieu-Denoncourt et al. 2015; Miodovnik et al. 2014; U.S. Consumer Product Safety Commission 2014).

### Regrettable Substitution

Under our current system, when a toxic chemical or category of chemicals is finally removed from the market, chemical manufacturers often substitute similar chemicals that may pose similar concerns or be virtually untested for toxicity. This practice can result in “regrettable substitution” whereby the cycle of exposures and adverse effects starts all over again. The following list provides examples of this cycle:

When the federal government banned some uses of OP pesticides, manufacturers responded by expanding the use of neonicotinoid and pyrethroid pesticides. Evidence is emerging that these widely used classes of pesticides pose a threat to the developing brain (Kara et al. 2015; Richardson et al. 2015; Shelton et al. 2014).
When the U.S. Government reached a voluntary agreement with flame retardant manufacturers to stop making PBDEs, the manufacturers substituted other halogenated and organophosphate flame retardant chemicals. Many of these replacement flame retardants are similar in structure to other neurotoxic chemicals but have not undergone adequate assessment of their effects on developing brains.
When the federal government banned some phthalates in children’s products, the chemical industry responded by replacing the banned chemicals with structurally similar new phthalates. These replacements are now under investigation for disrupting the endocrine system.

### Looking Forward

Our system for evaluating scientific evidence and making decisions about environmental chemicals is broken. We cannot continue to gamble with our children’s health. We call for action now to prevent exposures to chemicals and pollutants that can contribute to the prevalence of neurodevelopmental disabilities in America’s children.

We need to overhaul our approach to developing and assessing evidence on chemicals of concern for brain development. Toward this end, we call on regulators to follow scientific guidance for assessing how chemicals affect brain development, such as taking into account the special vulnerabilities of the developing fetus and children, cumulative effects resulting from combined exposures to multiple toxic chemicals and stressors, and the lack of a safety threshold for many of these chemicals (Committee on Improving Analysis Approaches Used by the U.S. EPA 2009). We call on businesses to eliminate neurodevelopmental toxicants from their supply chains and products, and on health professionals to integrate knowledge about environmental toxicants into patient care and public health practice.

Finally, we call on policy makers to take seriously the need to reduce exposures of all children to lead—by accelerating the clean up from our past uses of lead such as in paint and water pipes, by halting the current uses of lead, and by better regulating the industrial processes that cause new lead contamination.

We are confident that reducing exposures to chemicals that can interfere with healthy brain development will help to lower the prevalence of neurodevelopmental disabilities, and thus enable many more children to reach their full potential.
